# Exercise type–brain region interactions determine the effects of exercise on the hippocampus

**DOI:** 10.3389/fnins.2025.1728835

**Published:** 2026-01-12

**Authors:** Ryuki Tsuchida, Taisei Yamaguchi, Ryunosuke Naochi, Daisuke Funabashi, Takeshi Nishijima

**Affiliations:** 1 Department of Human Health Sciences, Graduate School of Human Health Sciences, Tokyo Metropolitan University, Hachioji, Japan; 2 Japan Society for the Promotion of Science (JSPS) Research Fellowship for Young Scientists, Tokyo, Japan; 3 Faculty of Education, Hakuoh University, Oyama, Japan

**Keywords:** antidepressant effect, exercise type, hippocampus, neural activity, neurogenesis

## Abstract

**Introduction:**

Exercise enhances hippocampal function, which is critical for learning, memory, and dementia prevention. However, the effects of exercise depend not only on intensity and frequency but also on the type of exercise, and not all exercise paradigms reliably activate hippocampal circuits.

**Methods:**

We compared treadmill exercise (TE) and rotarod exercise (RE) in mice to determine whether exercise type differentially influences neural activity and hippocampal plasticity across brain regions, using acute and chronic exercise paradigms.

**Results:**

In acute exercise experiments, TE robustly increased neuronal activation in the dorsal hippocampus and entorhinal cortex. In contrast, RE activated other brain regions, including the ventral hippocampus and dorsal raphe nucleus, but did not increase activity in the dorsal hippocampus–entorhinal pathway. In chronic phase experiments, both TE and RE produced antidepressant effects, whereas only TE stimulated hippocampal neurogenesis.

**Discussion:**

These results demonstrate that the effects of exercise on the brain are determined by interactions between exercise type and brain region. The findings highlight region-specific and exercise type-dependent characteristics of exercise-induced brain plasticity, underscoring the importance of considering exercise type when aiming to promote hippocampal health and prevent dementia.

## Introduction

The hippocampus is a region integral to learning and memory and may thereby mitigate the onset and progression of dementia (Choi et al., 2018; Llorens-Martín, 2018; Horowitz et al., 2020). Extensive evidence has indicated that physical exercise exerts positive effects on hippocampal function (Rendeiro and Rhodes, 2018; Aghjayan et al., 2021). Exercise influences multiple neurophysiological processes in the hippocampus, including increasing neuronal activity (Lee et al., 2003; Clark et al., 2011), enhancing cerebral perfusion (Nishijima and Soya, 2006; Nishijima et al., 2012), and upregulating neurotrophic factors such as brain-derived neurotrophic factor (BDNF) (Vaynman et al., 2004; Soya et al., 2007b; Erickson et al., 2012; Alomari et al., 2013). These effects are reinforced in the long-term by promoting hippocampal neurogenesis (Van Praag et al., 1999; Van Praag, 2008; Nishii et al., 2017), increasing hippocampal volume (Erickson et al., 2011; Chaddock et al., 2010) and ultimately enhancing cognitive performance (Erickson et al., 2011; Van Praag et al., 1999; Cotman and Berchtold, 2002; Berchtold et al., 2010; Chaddock et al., 2010; da Silva de Vargas et al., 2017). Moreover, the neuroscientific mechanisms underlying exercise-induced enhancement of hippocampal function have been elucidated (Rendeiro and Rhodes, 2018; Aghjayan et al., 2021).

The effects of exercise on the hippocampus are known to be influenced by factors such as exercise intensity and frequency. Recently, growing attention has been directed toward the differential impact of exercise types, including open- versus closed-skill sports, aerobic versus resistance training, and indoor versus outdoor exercise, on cognitive function (Gökçe et al., 2021; Boere et al., 2023; Walters et al., 2025). However, no consistent conclusions have been drawn regarding the most effective type of exercise. Several studies have suggested that certain exercise types may exert only limited or negligible effects on dementia prevention (Sink et al., 2015; De Souto Barreto et al., 2018; Vints et al., 2024; Yuan et al., 2024). Importantly, differential effects of these exercise types have also been reported in the hippocampus (Nokia et al., 2016; Tsuchida et al., 2022; Jiang et al., 2024; Bloch-Ibenfeldt et al., 2025). These inconsistencies highlight the critical need to refine our understanding of how specific exercise modalities shape hippocampal function. Our previous study also showed that certain types of exercise fail to activate hippocampal neural activity in mice (Tsuchida et al., 2022). In that study, we compared the effects of treadmill exercise (TE), one of the most used exercise types in rodents, with those of rotarod exercise (RE), commonly used to assess motor learning and coordination, on hippocampal neural activity. TE and RE were chosen because they allow precise matching of exercise intensity and duration, a key requirement for directly comparing different exercise types. As a result, only TE was found to activate neural activity in the hippocampus, despite matched exercise intensity. These findings indicate that the effect of exercise on the hippocampus depends on exercise type, with some modalities failing to reliably induce neural activation in the hippocampus. While highlighting exercise type as a key determinant of hippocampal responsiveness, the results further raise the question of whether the lack of activation observed with RE is hippocampus-specific or it can be observed in other brain regions as well.

To address this knowledge gap, we conducted two experiments to compare TE and RE, focusing on their differential effects on neural activity across multiple brain regions. Specifically, we investigated whether the limited impact of RE was confined to the hippocampus or extended to other brain structures. Our findings corroborate those of previous reports, demonstrating that RE does not enhance neural activity in the dorsal hippocampus but can promote activity in other regions. By clarifying that the effects of exercise on the brain depend on the interaction between exercise type and brain region, our results offer new insights into why certain exercise modalities are less effective for improving hippocampal functions and inform the development of targeted exercise-based interventions for dementia prevention.

## Materials and methods

### Animals

Four-weeks-old adult male C57BL/6J mice (*n* = 40 for Exp. 1, *n* = 32 for Exp. 2) were purchased (SLC, Shizuoka, Japan) and housed in groups of five per cage under controlled conditions. Mice were housed under a controlled temperature (22 ± 2 °C) and a 12-h light/dark cycle (lights on at 2:00), with *ad libitum* access to food and water. All experiments were performed during the early dark phase, between 14:30 and 16:30. All experimental procedures were approved by the Animal Experimental Ethics Committee of Tokyo Metropolitan University (Approval No. A7-022) and conducted in accordance with the ARRIVE (Animal Research: Reporting of *In Vivo* Experiments) guidelines. All efforts were made to minimize animal suffering. All experimental protocols complied with relevant institutional and national guidelines and regulations.

### Exercise protocol

Experiment 1: At 8 weeks of age, the mice were divided into two groups: TE (*n* = 20) and RE (*n* = 20). Mice in the TE and RE groups were respectively, habituated to a treadmill (KN-73, Natsume, Tokyo, Japan) and a rotarod apparatus (47650 Rota-Rod NG, Ugo-Basile, Varese, Italy) for 30 min/day for 5 days. The exercise protocols followed our previous study (Tsuchida et al., 2022), which established matched-intensity treadmill and rotarod exercise. To ensure that the two exercise types were compared at equivalent physiological loads, we determined exercise intensity based on lactate threshold (LT). When exercise intensity is gradually increased, blood lactate levels do not increase during low-intensity exercise and begin to rise sharply only once the exercise intensity exceeds the LT (Von Duvillard, 2001). Based on this physiological principle and our previous study (Tsuchida et al., 2022), together with relevant previous literature (Soya et al., 2007a; Okamoto et al., 2015), we standardized the exercise intensity for both treadmill and rotarod exercises to a level just below the LT (TE for 15 m/min and RE for 30 rpm). During the habituation sessions for each exercise, the exercise speed was gradually increased, such that on the last day, mice in the treadmill group were able to run at a speed of 15 m/min, and those in the rotarod group were able to walk on the rotarod at 30 rpm for 30 min. A slight electrical shock was occasionally applied to motivate running on the treadmill, and mice that fell from the rotarod were immediately placed back onto the rod. On the test day, however, mice rarely received shocks on the treadmill, and only a few mice fell from the rotarod. Following the last exercise session, mice were allowed to rest for 2 days before the test to eliminate any residual effects of the habituation sessions on neural activity, as c-Fos expression is transient and returns to basal levels within 6 h (Barros et al., 2015). On the day of the experiment, the mice were randomly assigned to one of the following groups (10 mice per group): TE (15 m/min), TC (0 m/min), RE (30 rpm), or RC (0 rpm). Mice in the TE and RE groups were subjected to 30 min of exercise at the indicated speeds. Mice in the control groups were placed on a stationary treadmill or rotarod for 30 min. The mice in the control groups also underwent habituation sessions.

Experiment 2: experimental groups were the same as those in Experiment 1. The mice were subjected to 5 weeks of exercise training, five times per week for 30 min each time. During the exercise period, the speed was gradually increased to 15 m/min for TE and 30 rpm for RE, reaching these intensities within the first 5 days and maintained thereafter. The mice in the control groups were placed on a stationary treadmill or rotarod for the same duration. Each behavioral test was conducted after 4 weeks of exercise.

### Forced swim test

The test was conducted over 2 days. A 6-min forced swim session was conducted on day 1 to allow the mice to feel a sense of inescapability, followed by a 6-min forced swim test (FST) 24 h later. Mice were individually placed in a Plexiglas cylinder (12 cm diameter, 24 cm height) containing 19 cm of water (24 °C ± 1 °C) and were videorecorded for 6 min. Immobility was scored during the last 5 min of the test. Immobility was defined as the absence of movement, except for that required to keep the head above water. The analysts were blinded to the experimental groups.

### Morris water maze

The Morris water maze (MWM) is divided into a learning phase, which assesses spatial learning, and a probe test, which assesses spatial memory. This test was performed in a circular pool measuring 1200 mm in diameter and 300 mm in depth (24 °C ± 1 °C), containing a hidden platform 100 mm in diameter placed at the center of one quadrant. Visual cues were placed around the pool for the mice to acquire spatial information during both the learning period and the probe test. During the 4-days learning phase, the mice searched for the position of the platform for 60 s (two trials per day); if unsuccessful, they were manually guided to it. After reaching the platform, they remained there for 15 s, and the time taken to reach the platform was measured. One day after the final learning session, the platform was removed, and a probe trial was conducted. The mice explored the pool for 60 s, and the time spent in the quadrant previously containing the platform was recorded. Recordings were performed using a video camera, and the results were quantified using the video tracking system software ANY-MAZE (Stoelting Co.).

### c-Fos immunohistochemical staining

Ninety minutes after the acute bout of exercise ended, the mice were deeply anesthetized with sodium pentobarbital (100 mg/kg body weight, intraperitoneal injection) and then euthanized via transcardial perfusion with cold saline. Depth of anesthesia was confirmed by the absence of reflex responses to paw pinch prior to perfusion. The mice brains were rapidly removed and fixed in 4% paraformaldehyde in phosphate-buffered saline (PBS, pH 7.4) for two nights. The specimens were then cryoprotected with 30% sucrose in PBS and frozen. Brains were sectioned using a freezing microtome (REM-710, Yamato Kohki Industrial, Saitama, Japan) to obtain coronal sections (40-μm thickness) of the dorsal hippocampus (anteroposterior −2.18 to −1.34 mm from the bregma), entorhinal cortex (anteroposterior −4.16 to −3.40 mm from the bregma), ventral hippocampus (anteroposterior −3.80 to −3.16 mm from the bregma), and 5-HT neurons in the dorsal raphe nucleus (DRN; anteroposterior −4.84 to −4.24 mm from the bregma) according to the mouse brain atlas. Sections were stored in PBS containing 0.01% sodium azide.

Immunohistochemistry for c-Fos and 5-HT was performed as described in our previous study, with minor modifications (Morikawa et al., 2021; Tsuchida et al., 2022). Free-floating sections were pre-incubated with 1% H2O2 to quench endogenous peroxidase activity. After rinsing in PBS with 0.5% Triton X-100 and 0.5% bovine serum albumin (PBT-BSA), the sections were incubated with rabbit monoclonal anti-c-Fos antibody (1:5,000, #2250, Cell Signaling Technology, Danvers, MA, USA) diluted in PBT-BSA for 48 h at 4 °C. The sections were then incubated with a biotinylated secondary anti-rabbit IgG antibody (1:1,000, #AP182B, EMD Millipore, Burlington, MA, USA) diluted in PBT-BSA overnight at 4 °C. Sections were then treated with an avidin-biotin-peroxidase complex (Vectastain ABC Peroxidase Kit, Vector Laboratories, Burlingame, CA, USA) for 2 h at room temperature. Finally, the antigens were visualized with 0.02% 3,3-diaminobenzidine (DAB) in a 0.1 M Tris-HCl solution (pH 7.6) containing nickel ammonium sulfate and 0.001% H2O2. c-Fos immunoreactivity was localized to the cell nuclei, appearing as a dark gray-black stain. For dual immunostaining of 5-HT, the sections were sequentially incubated with 5-HT antibody (1:1,000, 20080, OriGene Technologies, Rockville, MD, USA) for 48 h at 4 °C. Avidin-biotin-peroxidase complexes were visualized using DAB in a 0.1 M Tris-HCl buffer without nickel sulfate. Moreover, 5-HT immunoreactivity was localized in the cell cytoplasm and was visible as light-brown staining. The sections were mounted on gelatin-coated slides, dehydrated in a graded ethanol series, cleared in xylene, and coverslipped. The slides were coded randomly to ensure unbiased analysis.

### Quantification of c-Fos-positive cells

To calculate the density of c-Fos-immunoreactive (c-Fos-ir) cells in each brain region, digital images were acquired using an optical microscope (×20 objective lens, BX-53, Olympus, Shinjuku City, Japan). The surface areas of the brain regions were measured using image analysis software (Image J, Bethesda, MD, USA). Next, the number of c-Fos-ir cells in each region was manually counted, and the density of c-Fos-ir cells was determined. This procedure was repeated for six to eight sections per mouse, and the mean cell density was calculated for each animal.

### DCX immunohistochemical staining

At the end of the Experiment 2, mice were deeply anesthetized with sodium pentobarbital (100 mg/kg body weight, intraperitoneal injection) and then euthanized via transcardial perfusion with cold saline. Depth of anesthesia was confirmed by absence of reflex responses to paw pinch prior to perfusion. Their brains were rapidly removed and fixed in 4% paraformaldehyde in PBS (pH 7.4) for two nights. They were then cryoprotected with 30% sucrose in PBS and frozen. Brains were sectioned using a freezing microtome (REM-710, Yamato Kohki Industrial, Saitama, Japan) to obtain coronal sections (40-μm thickness) to encompass the whole hippocampus. Sections were stored in PBS containing 0.01% sodium azide.

Immunohistochemistry for doublecortin (DCX) was performed as previously described (Nishijima et al., 2017; Funabashi et al., 2021). Free-floating sections were pre-incubated with 1% H2O2 to quench endogenous peroxidase activity. After rinsing in PBT–BSA, sections were incubated with rabbit monoclonal anti-DCX antibody (1:1000, #4604, Cell Signaling Technology, Danvers, MA, USA) diluted in PBT-BSA for 48 h at 4 °C. The sections were then incubated with a biotinylated secondary anti-rabbit IgG antibody (1:1,000, #AP182B, EMD Millipore, Burlington, MA, USA) diluted in PBT-BSA overnight at 4 °C. Sections were then treated with an avidin-biotin-peroxidase complex (Vectastain ABC Peroxidase Kit, Vector Laboratories, Burlingame, CA, USA) for 2 h at room temperature. Finally, the antigens were visualized with 0.02% DAB in a 0.1 M Tris–HCl solution (pH 7.6) containing 0.001% H2O2. Cell nuclei were counterstained with Nissl. The sections were mounted on gelatin-coated slides, dehydrated in a graded ethanol series, cleared in xylene, and coverslipped. The slides were coded randomly to ensure unbiased analysis.

### Quantification of hippocampal neurogenesis

DCX-immunoreactive immature neurons were quantified as previously described (Nishijima et al., 2017; Funabashi et al., 2021). DCX-ir immature neurons overlap in 40 μm thick sections, making it difficult to accurately count their somata. Therefore, we previously proposed a new approach for counting DCX-ir immature neurons based on their morphological characteristic of having, on average, a single dendrite when measured within 40 μm of their soma (Revest et al., 2009). Briefly, digital images (1,920 × 1,440) of the dentate gyrus (DG) were acquired using a BX-53 microscope (×10 objective lens, Olympus) throughout the rostral/caudal region of the hippocampus. Using the ImageJ software (National Institutes of Health, Bethesda, MD, USA), a segmented line was drawn along the middle of the granule cell layer (GCL), and crossings over the dendrites of DCX-ir immature neurons were counted and divided by the length of the line. This was repeated for eight to ten images taken from six to eight sections per mouse, and the average was calculated for each animal. Because the thickness of the GCL is approximately 60–80 μm, the value thus obtained (crossings/mm) should reflect the density of DCX-ir immature neurons in the DG.

### Statistical analysis

All data, except escape latency in the acquisition phase of the MWM, were analyzed using a two-way repeated-measures analysis of variance (ANOVA). Escape latency in the acquisition phase of the MWM was analyzed using a three-way repeated-measures ANOVA. If a significant interaction or main effect was observed, Tukey’s *post-hoc* test was used to assess statistical differences between the groups. Data are expressed as mean ± standard error of the mean. The threshold for statistical significance was set at *p* < 0.05.

## Results

### Experiment 1: effects of the different types of acute exercises on neuronal activity across multiple brain regions

Our previous studies demonstrated that the effects of an acute bout of exercise on hippocampal neuronal activation are influenced by the exercise type (Tsuchida et al., 2022). Specifically, TE activated hippocampal neural activity, whereas RE failed to elicit comparable activation. However, in our previous study, we did not examine brain regions other than the dorsal hippocampus. Therefore, it remains unclear whether this lack of activation is specific to the hippocampus or whether RE has any impact on other brain regions.

**FIGURE 1:**
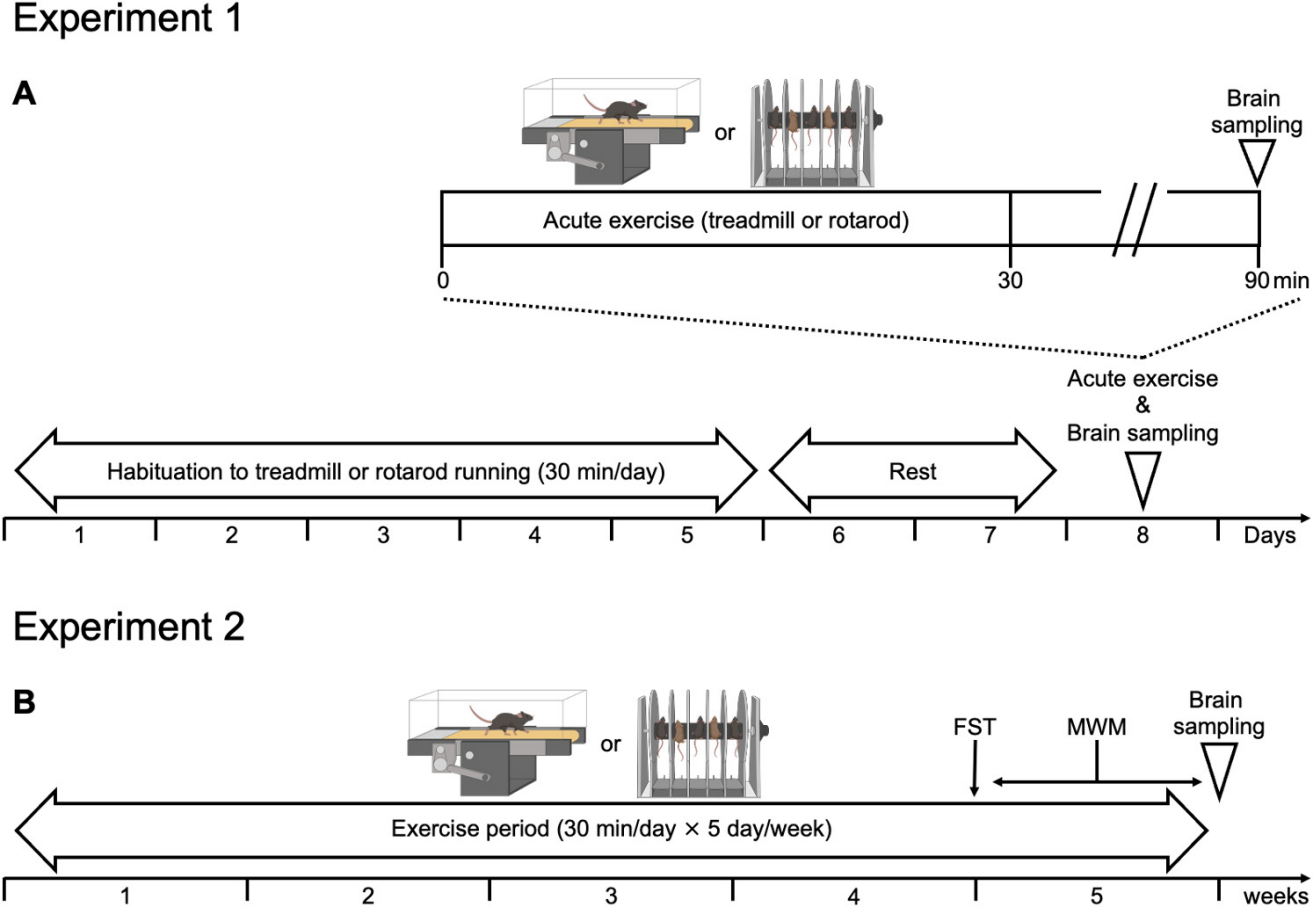
Experimental design. **(A)** Experimental 1. Effects of the different types of exercise on neuronal activity in each brain regions. After acclimation to each exercise apparatus, the mice were assigned to four groups (TC, treadmill control; TE, treadmill exercise; RC, rotarod control; RE, rotarod exercise) and subjected to an acute exercise session, followed by assessment of neural activity in selected brain regions. **(B)** Experimental 2. Chronic rotarod exercise does not promote hippocampal neurogenesis. Mice underwent repeated TE or RE for 5 weeks, after which behavioral tests and immunohistochemical analyses were conducted to evaluate chronic effects.

In this experiment, we focused on the dorsal hippocampus and also examined the ventral hippocampus, the entorhinal cortex, and serotonergic (5-HT) neurons in the DRN. The dorsal and ventral hippocampus were assessed because they are functionally dissociable, with the dorsal region being critical for spatial learning and memory (Moser and Moser, 1998) and the ventral region being involved in emotion and stress regulation (Bannerman et al., 2004). The entorhinal cortex was included as it serves as a major cortical input to the hippocampus and is essential for hippocampal information processing (Zhang et al., 2014). Finally, we examined 5-HT neurons in the DRN because this region is deeply implicated in depression, and exercise exerts antidepressant effects, partly through the modulation of the serotonergic system (Lowry et al., 2005; Greenwood and Fleshner, 2011). Although TE has been reported to activate neuronal activity in the dorsal and ventral hippocampus and 5-HT neurons in the DRN, it remains unknown how rotarod exercise affects them (Lee et al., 2003; Otsuka et al., 2016; Nishii et al., 2017; Tsai et al., 2019; Tsuchida et al., 2022). The experimental design is shown in Figure 1A. Following our previous study (Tsuchida et al., 2022), the intensities of both exercises were matched just below the LT, with the TE for 15 m/min and the RE for 30 rpm. Mice were subjected to 30 min of exercise, after which brain neural activity was assessed using c-Fos immunohistochemistry.

**FIGURE 2:**
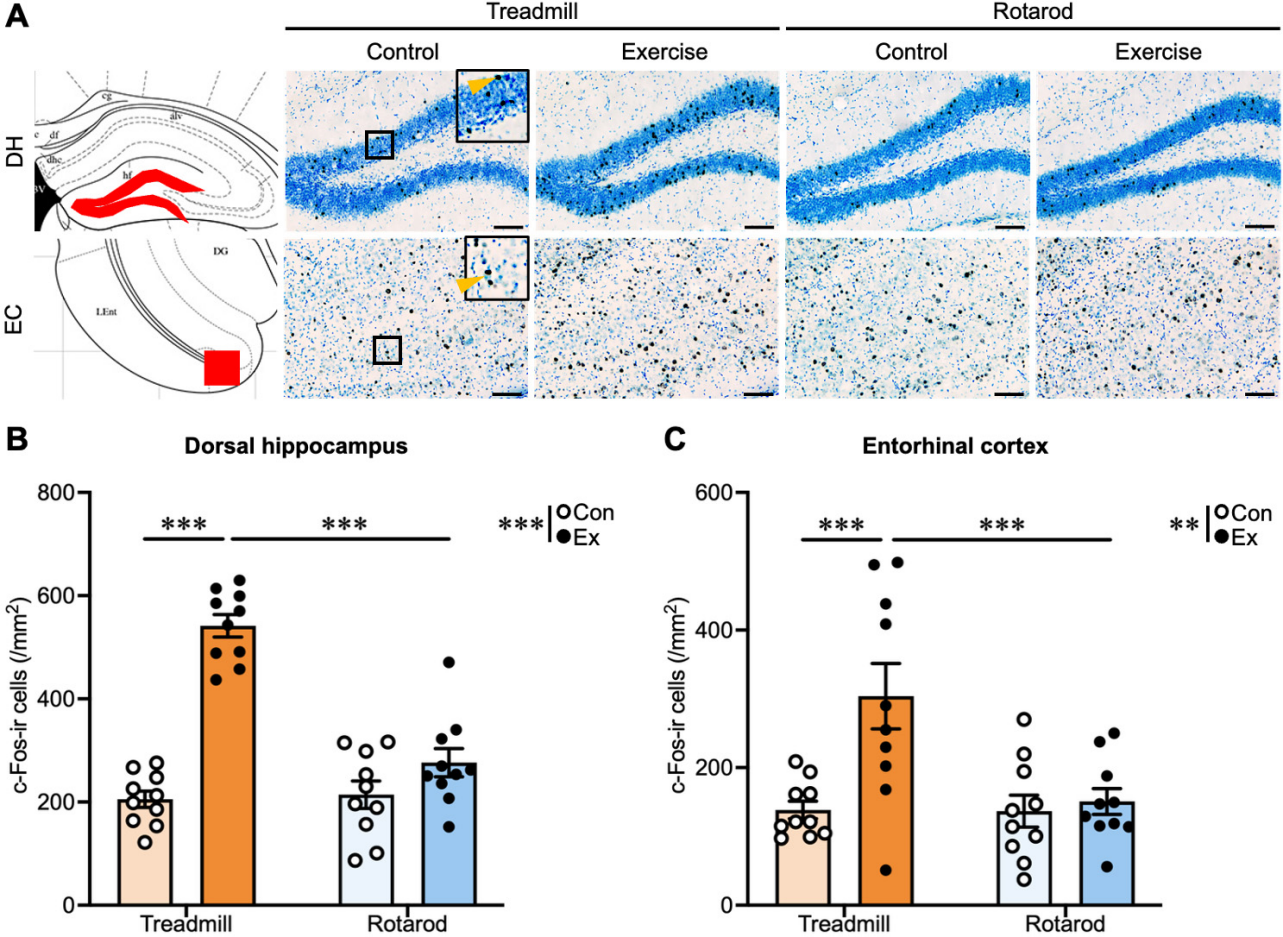
Effects of the different types of exercise on neuronal activity across the brain regions. **(A)** Representative images of c-Fos-ir cells in the dorsal hippocampus and entorhinal cortex. Scale bar: 100 μm. Insets show enlarged views of the boxed regions. Arrows indicate representative cells that were identified as c-Fos-ir cells. **(B)** The density of c-Fos-ir cells in the dorsal hippocampus. **(C)** The density of c-Fos-ir cells in the entorhinal cortex. Treadmill control (TC, light orange bars), treadmill exercise (TE, orange), rotarod control (RC, light blue) and rotarod exercise (RE, blue) mice (*n* = 10 per group). Values represented as mean ± SEM. Statistical significance was calculated using two-way analysis of variance (ANOVA) and Tukey’s *post-hoc* tests (**P < 0.01, ***P < 0.001).

**FIGURE 3:**
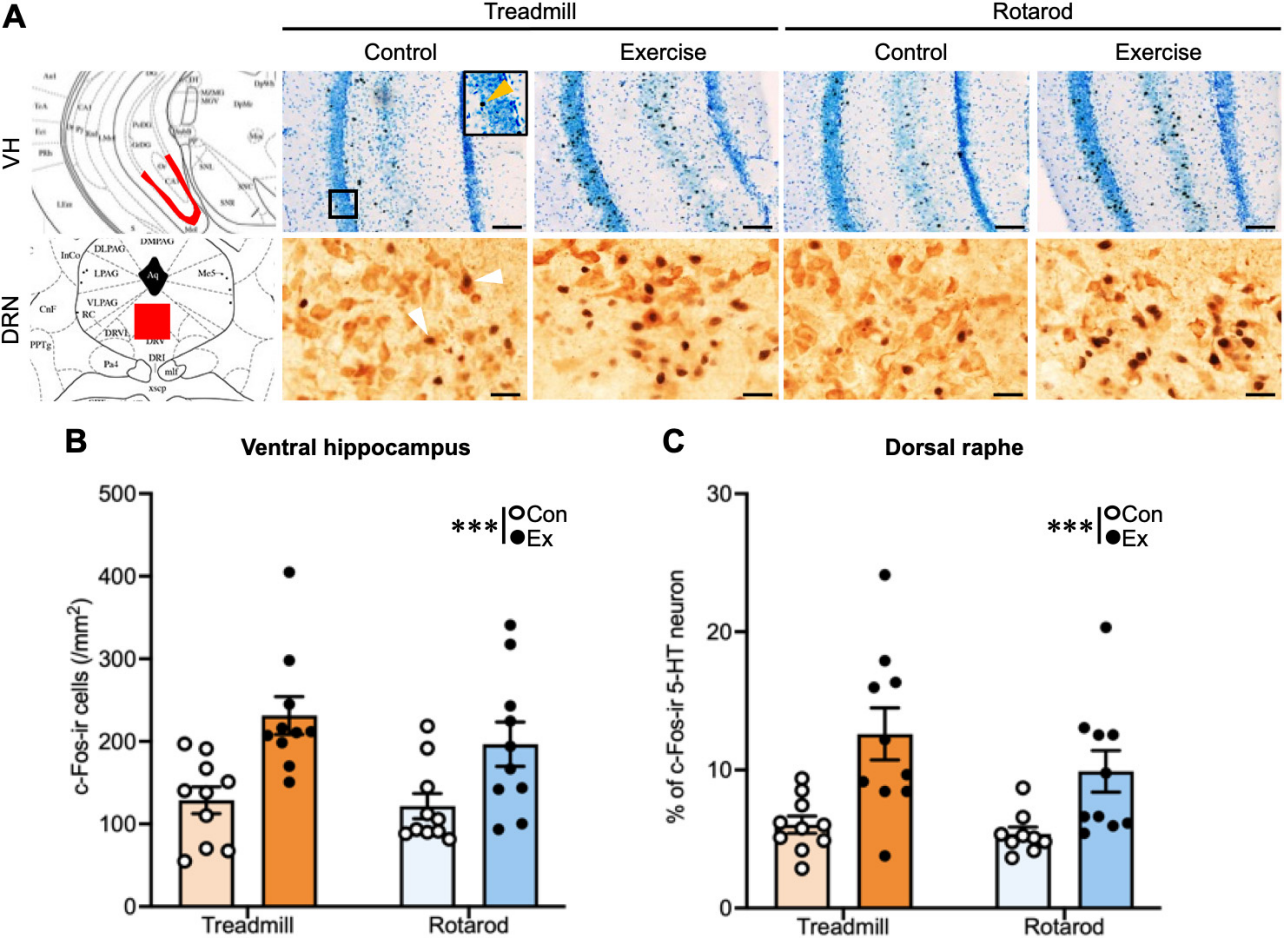
Effects of different types of exercise on neuronal activity across the brain regions. **(A)** Representative images of c-Fos-ir cells in the ventral hippocampus and 5-HT neurons in the DRN. Scale bar: 100 μm (ventral hippocampus), 20 μm (5-HT neurons in the DRN). Insets show enlarged views of the boxed regions. Arrows indicate representative cells that were identified as c-Fos-ir cell in ventral cortex or double-labeled cells (c-Fos/5-HT) in DRN. **(B)** The density of c-Fos-ir cells in the ventral hippocampus. **(C)** The density of c-Fos-ir cells in 5-HT neurons in the DRN. *n* = 10 per group. Values represented as mean ± SEM. Statistical significance was calculated using two-way ANOVA and Tukey’s *post-hoc* tests (***P < 0.001).

Representative images of immunostaining are shown in Figures 2A, 3A. In the dorsal hippocampus, a two-way ANOVA revealed significant main effects of exercise [*F*(1, 36) = 34.63, *P* < 0.001], type [*F*(1, 36) = 30.25, *P* < 0.001], and an exercise × type interaction [*F*(1, 36) = 73.22, *P* < 0.001]. *Post hoc* analysis indicated that the density of c-Fos-immunoreactive (c-Fos-ir) cells in the dorsal hippocampus was increased in the TE group (*P* < 0.001), but not in the RE group, compared to the respective controls (Figure 2B), which is consistent with our previous findings (Tsuchida et al., 2022). Similarly, in the entorhinal cortex, as in the dorsal hippocampus, significant main effects of exercise [*F*(1, 36) = 9.65, *P* < 0.01], type [*F*(1, 36) = 7.22, *P* < 0.05], and an exercise × type interaction [*F*(1, 36) = 6.90, *P* < 0.05] were observed. *Post-hoc* tests showed that the density of c-Fos-ir cells increased only in the TE group (*P* < 0.001), but not in the RE group (Figure 2C).

In contrast, in the ventral hippocampus, a two-way ANOVA revealed a significant main effect of exercise [*F*(1, 36) = 18.10, *P* < 0.001], but no significant effect of type [*F*(1, 36) = 1.00, *P* = 0.32] or exercise × type interaction [*F*(1, 36) = 0.44, *P* = 0.51]. Similarly, in 5-HT neurons of the DRN, a significant main effect of exercise [*F*(1, 36) = 18.20, *P* < 0.001] was observed, with no significant main effect of type [*F*(1, 36) = 1.68, *P* = 0.20] or interaction [*F*(1, 36) = 0.61, *P* = 0.44]. In these two brain regions, TE and RE groups demonstrated a comparable and significant increase in the density of c-Fos-ir cells (Figure 3).

In summary, consistent with our previous reports (Tsuchida et al., 2022), only TE increased neuronal activation in the dorsal hippocampus. A comparable enhancement pattern was observed in the entorhinal cortex, which contributes to hippocampal information processing. In contrast, the RE did not elicit comparable activation in these areas. These findings indicate that despite activating neural activity in some brain regions such as the ventral hippocampus and 5-HT neurons in the DRN, RE is ineffective in activating the dorsal hippocampus or entorhinal cortex. In our previous studies, we showed that the effects of exercise on the dorsal hippocampus differ depending on the type of exercise. The present experiment extends these findings by demonstrating that the impact of exercise on neuronal activation not only depends on the exercise type but also varies across the brain regions, highlighting an interaction between exercise type and brain regions. However, it remains unclear whether these effects of acute exercise translate into chronic adaptations. Therefore, we next examined whether chronic exercise of different types differentially affects hippocampal neurogenesis and behavioral outcomes. In addition, we assessed the antidepressant effects by investigating brain regions beyond the hippocampus, where exercise-induced effects have been recognized, focusing on regions deeply involved in mood regulation, such as the ventral hippocampus and serotonergic neurons in the DRN.

### Experiment 2: effects of the different types of chronic exercise on the hippocampus and antidepressant effect

Chronic exercise, including TE, is well established to enhance hippocampal neurogenesis and improve hippocampus-dependent functions (Van Praag et al., 1999; Cotman and Berchtold, 2002; Van Praag, 2008; Liu et al., 2009; Berchtold et al., 2010). However, Exp. 1 demonstrated an interaction between exercise type and brain region, showing that while TE activated both the dorsal and ventral hippocampus, entorhinal cortex, and 5-HT neurons in DRN, RE failed to induce neuronal activation in the dorsal hippocampus and entorhinal cortex. Based on this interaction, we hypothesized that long-term adaptations of the hippocampus would likewise depend on the exercise type, with chronic RE failing to enhance hippocampal neurogenesis or hippocampus-dependent functions. Additionally, because 5-HT neurons in the DRN, which are implicated in antidepressant actions, are activated by both TE and RE, we hypothesized that both types of exercise would exert antidepressant-like effects.

**FIGURE 4:**
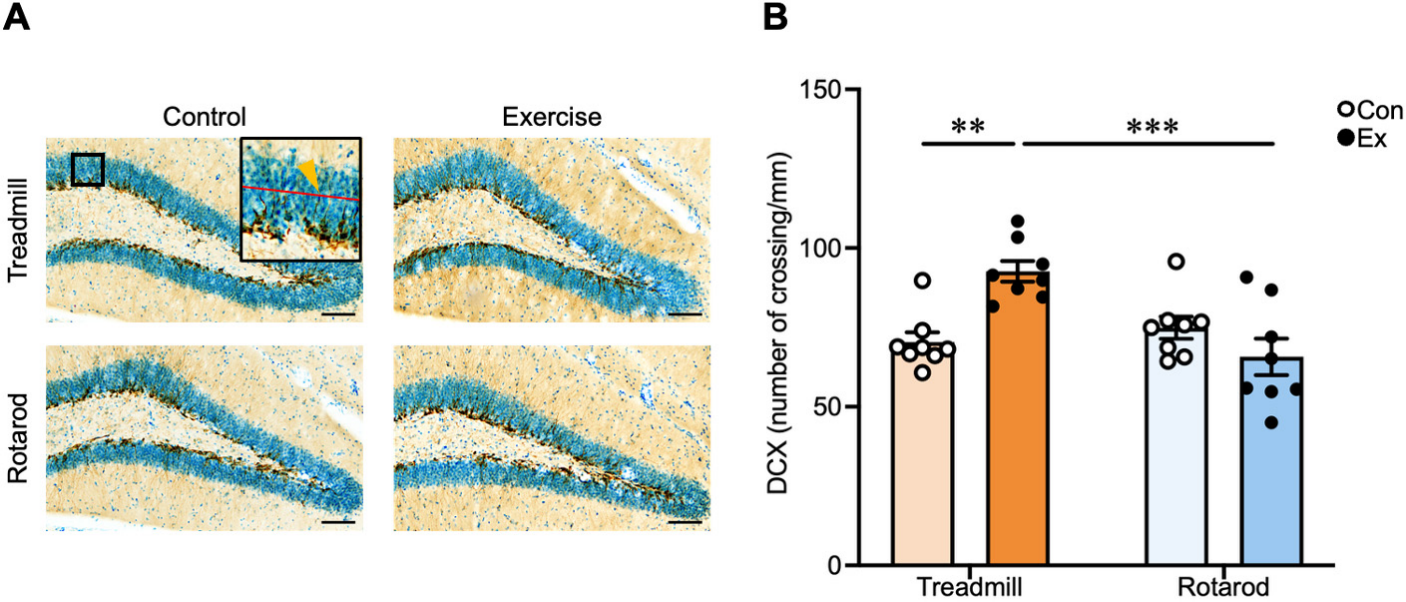
Chronic rotarod exercise does not promote hippocampal neurogenesis. **(A)** Representative images of DCX-ir neurons in the hippocampal dentate gyrus. Scale bar: 100 μm. Insets show enlarged views of the boxed regions. Arrows indicate representative cells that were identified as DCX-ir immature neuron. **(B)** The density of DCX-ir immature neurons in the dorsal hippocampal dentate gyrus (*n* = 8 per group). Values represented as mean ± SEM. Statistical significance was calculated using two-way ANOVA and Tukey’s *post-hoc* tests (**P < 0.01, ***P < 0.001).

The experimental design is shown in Figure 1B. We subjected mice to either TE or RE for 5 weeks. In the 5th week, the FST and MWM were conducted to assess depression-like behavior and spatial learning and memory, respectively. MWM was conducted 2 days after the FST. This interval was introduced because FST can impose acute stress and physical load that may transiently influence subsequent spatial learning, particularly at the beginning of MWM, and the 2-days rest period minimized such adverse effects. Hippocampal neurogenesis was assessed using DCX immunohistochemistry (Nishijima et al., 2017; Funabashi et al., 2021). Representative images of immunostaining are shown in Figure 4A. A two-way ANOVA revealed a significant main effect of type [*F*(1, 28) = 7.69, *P* < 0.01] and exercise × type interaction [*F*(1, 28) = 15.1, *P* < 0.001] but no significant main effect of exercise [*F*(1, 28) = 2.65, *P* = 0.11]. *Post hoc* analysis revealed that the density of immature DCX-immunoreactive (DCX-ir) neurons was significantly higher in the TE (*P* < 0.01), but not in the RE, indicating that hippocampal neurogenesis was enhanced only in the TE group (Figure 4). Our finding that chronic TE promotes hippocampal neurogenesis is consistent with those of previous reports (Uda et al., 2006; Okamoto et al., 2012, 2015; Inoue et al., 2015; Nishii et al., 2017). In contrast, the absence of a comparable effect after RE suggests that not all types of exercise promote hippocampal neurogenesis.

**FIGURE 5:**
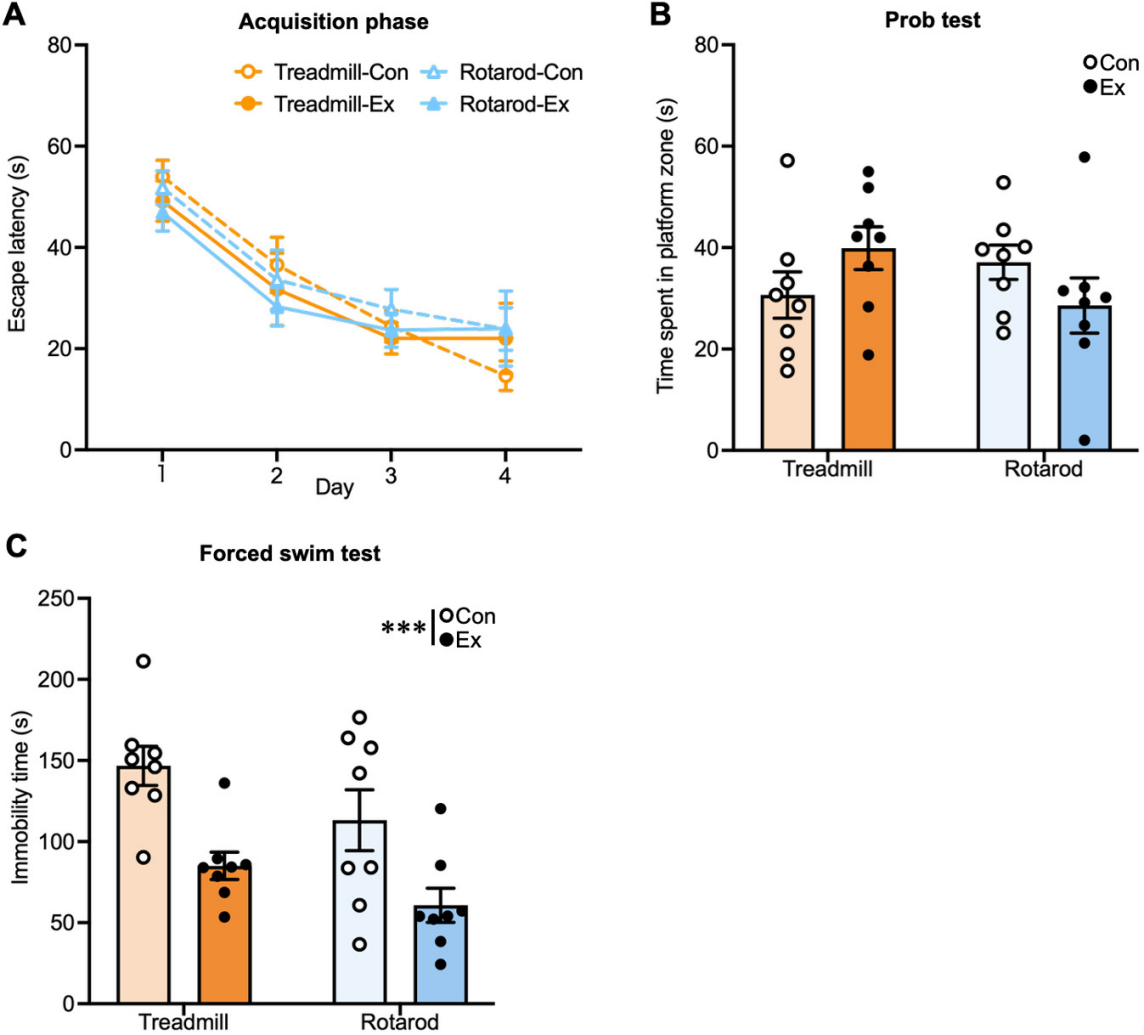
Effects of the different types of chronic exercise on the hippocampus and the antidepressant effect. **(A–C)** Behavioral test results (*n* = 8 per group). **(A)** Escape latency in the acquisition phase of the Morris water maze (MWM). Values represented as mean ± SEM. Statistical significance was calculated using three-way ANOVA. **(B)** Time spent in the platform zone in the probe test of MWM. **(C)** Immobility time in the forced swim test. Values represented as mean ± SEM. Statistical significance was calculated using two-way ANOVA and Tukey’s *post-hoc* tests (***P < 0.001).

In the current experiment, although positive effects of chronic TE on MWM performance were expected, we did not observe such effects in either the acquisition phase or the probe test (Figures 5A, B). In the acquisition phase (Figure 5A), a three-way ANOVA revealed a significant main effect of day [*F*(3, 84) = 37.34, *P* < 0.001], indicating learning across sessions. However, no significant main effects of exercise [*F*(1, 28) = 0.05, *P* = 0.82] or type [*F*(1, 28) = 0.66, *P* = 0.42] were detected. Moreover, there were no significant interactions among day, exercise, and type [day × exercise × type: *F*(3, 84) = 0.15, *P* = 0.93; exercise × type: *F*(1, 28) = 0.17, *P* = 0.68; day × type: *F*(3, 84) = 0.93, *P* = 0.43; day × exercise: *F*(3, 84) = 0.91, *P* = 0.44]. Similarly, in the probe test (Figure 5B), a two-way ANOVA revealed no significant main effects of exercise [*F*(1, 28) = 0.007, *P* = 0.94] or type [*F*(1, 28) = 0.30, *P* = 0.59], and no significant exercise × type interaction [*F*(1, 28) = 3.96, *P* = 0.06]. These results indicate that neither TE nor RE improved spatial learning or memory performance under the present experimental conditions. The non-significant differences in MWM performance may reflect the combination of a ceiling effect in young adult mice and the presence of strong visual cues that allowed all groups to learn the platform location efficiently, rather than the true absence of exercise-induced hippocampal adaptation. In particular, young adult mice demonstrate learning in the MWM even without exercise, which may limit the observable positive effect of exercise on spatial learning and memory (De Fiebre et al., 2006; Wang and Wang, 2016).

Finally, chronic TE reduced the immobility time in the FST compared with the respective controls (Figure 5C), replicating the antidepressant effect of TE (Nishii et al., 2017). Similarly, RE reduced the immobility time (Figure 5C), consistent with the observed activation of 5-HT neurons following RE, indicating an antidepressant effect. A two-way ANOVA revealed significant main effects of exercise [*F*(1, 28) = 19.2, *P* < 0.001] and type [*F*(1, 28) = 4.94, *P* < 0.05], but no significant exercise × type interaction [*F*(1, 28) = 0.13, *P* = 0.73], indicating that antidepressant effects are independent of exercise type.

Experiments 1 and 2 demonstrate that exercise type modulates the impact of exercise on the hippocampus across both acute and chronic timescales. RE has no promotive effect on hippocampal plasticity at either acute or chronic timescales, although it exerts beneficial effects on neuronal activity in the ventral hippocampus and 5-HT neurons as well as antidepressant outcomes. These findings highlight the dissociation between the role of exercise in hippocampal plasticity and its broad impact on emotional and depression-related brain regions. Taken together, our findings suggest that the lack of beneficial effects of RE is specific to the entorhinal–dorsal hippocampal pathway.

## Discussion

Our previous findings showed that TE activated dorsal hippocampal neural activity, whereas RE did not (Tsuchida et al., 2022). A key question was whether the lack of activation observed with RE was specific to the dorsal hippocampus or whether RE failed to induce neural activity in other brain regions. In this study, we investigated whether different types of exercise have distinct effects on neural activity across various brain regions and whether the lack of hippocampal activation during RE is specific to the dorsal hippocampus. We compared the TE and RE in both acute and chronic paradigms by examining the dorsal and ventral hippocampus, entorhinal cortex, and 5-HT neurons in the DRN. TE robustly activated the dorsal hippocampus and entorhinal cortex and promoted hippocampal neurogenesis, whereas RE failed to elicit comparable effects on neural activity in these regions or on hippocampal neurogenesis. In contrast, both exercise types activated other areas, including the ventral hippocampus and 5-HT neurons in the DRN, and produced antidepressant-like effects. Therefore, we observed that the lack of effect of RE was most evident in the dorsal hippocampus and entorhinal cortex, suggesting that the pathways connecting these regions may be less effectively engaged during RE.

Our findings highlight that the effects of exercise on the brain are determined not only by the presence or absence of exercise, but also by the interaction between exercise type and the brain region. Specifically, TE robustly activated the dorsal hippocampus and entorhinal cortex and promoted hippocampal neurogenesis, replicating previous findings (Van Praag et al., 1999; Lee et al., 2003; Van Praag, 2008; Inoue et al., 2015; Okamoto et al., 2015; Nishii et al., 2017; Tsuchida et al., 2022), whereas RE failed to elicit comparable effects in these regions (Figures 2, 4). Although RE is technically demanding and requires coordination and motor learning, it appears to be insufficient at inducing the neural activation required for hippocampal plasticity, highlighting the exercise type–specific nature of exercise-induced neurogenesis. Despite these differences in response within the hippocampus-entorhinal cortex, both TE and RE produced antidepressant-like effects, as evidenced by the reduced immobility in the FST (Figure 5). This behavioral outcome was paralleled by increased neural activity in the ventral hippocampus and the activation of 5-HT neurons in the DRN (Figure 3), indicating that the mood-related benefits of exercise can be mediated independently of hippocampal neurogenesis. These findings suggest that ventral hippocampal–serotonergic pathways serve as shared substrates underlying the antidepressant effects of diverse exercise types. Taken together, our results indicate that exercise engages neural circuits in a region- and exercise type-specific manner: TE preferentially recruits hippocampal networks supporting learning and memory, whereas RE primarily activates circuits implicated in mood regulation. These findings extend prior observations on exercise-type dependence (Sink et al., 2015; Nokia et al., 2016; De Souto Barreto et al., 2018; Gökçe et al., 2021; Tsuchida et al., 2022; Boere et al., 2023; Jiang et al., 2024; Vints et al., 2024; Yuan et al., 2024; Bloch-Ibenfeldt et al., 2025; Walters et al., 2025), underscore the importance of considering both exercise type and brain region in understanding the heterogeneous effects of exercise, and suggest that targeted exercise interventions could optimize neural and behavioral outcomes.

The key question was why RE failed to enhance hippocampal plasticity. Recently, peripheral factors released from the skeletal muscles, particularly myokines, have attracted attention as mediators of exercise-induced hippocampal plasticity. For example, irisin has been shown to enhance BDNF expression (Wrann et al., 2013) and stimulate cathepsin B to promote hippocampal neurogenesis (Moon et al., 2016), while lactate activates peroxisome proliferator-activated receptor-γ coactivator-1α–dependent BDNF signaling (Wrann et al., 2013; Park et al., 2021). In addition, systemic factors, such as insulin-like growth factor-1 (Trejo et al., 2001) and glycosylphosphatidylinositol-specific phospholipase D1 mainly secreted from the liver mediate the effects of exercise on hippocampal plasticity (Horowitz et al., 2020). Although we did not measure these peripheral factors, it is possible that they are not enhanced in RE. However, if RE also increases these peripheral factors, the absence of hippocampal activation and neurogenesis observed in the present study cannot be fully explained by those mechanisms alone. Whether these factors are induced by RE requires further investigation. Nevertheless, these findings suggest that although systemic factors are important mediators, the recruitment of specific neural circuits by distinct exercise types is also required to support hippocampal plasticity.

A critical consideration in interpreting our results is the difference in total running distance and speed between TE and RE. Although these differences could potentially influence neuronal activation, exercise volume alone does not fully account for our findings. Notably, despite the relatively lower running distance during RE, neural activation was still observed in regions such as the ventral hippocampus and 5-HT neurons in DRN. This indicates that RE was sufficient to drive neuronal activation in certain brain circuits. It is important to note that TE involves simple running, whereas RE requires coordinated, climbing-like movements, and therefore, equalizing speed or distance would not necessarily equate to equivalent physiological load. In our previous study, we standardized exercise intensity for both TE and RE to just below the LT, the point at which blood lactate sharply increases (Tsuchida et al., 2022). Taken together, these observations suggest that the effects of exercise on the brain are not determined solely by exercise volume, but rather by an interaction between exercise type and the specific brain regions engaged.

The present study has some limitations. First, this study only compared TE and RE; examining additional types of exercise, such as resistance training or outdoor running, would provide a broader understanding and help generalize the current findings. Second, neural activity was assessed only in selected brain regions, including the dorsal and ventral hippocampus, entorhinal cortex, and DRN. The effects of exercise on other cognition-related regions, such as the prefrontal cortex and amygdala, have not yet been examined. Finally, although the present findings suggest that the dorsal hippocampus and entorhinal cortex may be less effectively engaged during RE, further studies are required to elucidate the underlying mechanisms. Nevertheless, the present study provides a critical foundation for translating exercise-induced hippocampal activation into practical strategies, potentially informing future dementia prevention strategies and other clinical applications.

In conclusion, our results indicate that the effects of exercise on the brain are determined by the interaction between the exercise type and specific brain regions. RE elicited neural activation in the ventral hippocampus and 5-HT neurons but failed to enhance dorsal hippocampal and entorhinal cortex activity and hippocampal neurogenesis, whereas TE effectively promoted hippocampal plasticity across both acute and chronic timescales. These results underscore the region-specific, type-dependent nature of exercise-induced plasticity and highlight the critical importance of carefully tailoring exercise conditions to promote hippocampal health and prevent dementia.

## Data Availability

The raw data supporting the conclusions of this article will be made available by the authors, without undue reservation.
